# Surface roughness prediction of AISI D2 tool steel during powder mixed EDM using supervised machine learning

**DOI:** 10.1038/s41598-024-60543-3

**Published:** 2024-04-27

**Authors:** Amreeta R. Kaigude, Nitin K. Khedkar, Vijaykumar S. Jatti, Sachin Salunkhe, Robert Cep, Emad Abouel Nasr

**Affiliations:** 1 Symbiosis Institute of Technology, Symbiosis International (Deemed) University, Pune, 412115 India; 2 Department of Biosciences, Saveetha School of Engineering, Saveetha Institute of Medical and Technical Sciences, Chennai, India; 3 Faculty of Engineering, Department of Mechanical Engineering, Gazi University, Maltepe, Ankara, Turkey; 4 Department of Machining, Assembly and Engineering Metrology, Faculty of Mechanical Engineering, VSB-Technical University of Ostrava, 70800 Ostrava, Czech Republic; 5 Department of Industrial Engineering, College of Engineering, King Saud University, P.O. Box 800, 11421 Riyadh, Saudi Arabia

**Keywords:** Surface roughness, Jatropha oil, Linear regression, Decision tree, Random forest, Engineering, Mechanical engineering

## Abstract

Surface integrity is one of the key elements used to judge the quality of machined surfaces, and surface roughness is one such quality parameter that determines the pass level of the machined product. In the present study, AISI D2 steel was machined with electric discharge at different process parameters using Jatropha and EDM oil. Titanium dioxide (TiO_2_) nanopowder was added to the dielectric to improve surface integrity. Experiments were performed using the one variable at a time (OVAT) approach for EDM oil and Jatropha oil as dielectric media. From the experimental results, it was observed that response trends of surface roughness (SR) using Jatropha oil are similar to those of commercially available EDM oil, which proves that Jatropha oil is a technically and operationally feasible dielectric and can be efficiently replaced as dielectric fluid in the EDM process. The lowest value of S.R. (i.e., 4.5 microns) for EDM and Jatropha oil was achieved at current = 9 A, Ton = 30 μs, Toff = 12 μs, and Gap voltage = 50 V. As the values of current and pulse on time increase, the S.R. also increases. Current and pulse-on-time were the most significant parameters affecting S.R. Machine learning methods like linear regression, decision trees, and random forests were used to predict the surface roughness. Random forest modeling is highly accurate, with an R^2^ value of 0.89 and an MSE of 1.36% among all methods. Random forest models have better predictive capabilities and may be one of the best options for modeling complex EDM processes.

## Introduction

Electrical Discharge Machining (EDM) is an electrothermal and nonconventional machining process in which rapid and repetitive sparks are generated between the workpiece and tool, which are kept apart at a small gap of 0.001–0.5 mm and are submerged or flooded by dielectric fluid, by initiation of sparks controlled erosion of conductive material takes place^1^. Each spark produces enough heat to melt and evaporate material. The surface characteristics of the piece are significantly affected due huge amount of heat produced during the EDM operation. This phenomenon is inevitable but can be reduced by properly selecting machining parameters. Also, there are several methods to enhance EDM performance. One of the most widely used high-chromium and high-carbon steels in the D family, AISI D2 is distinguished by its strong wear resistance, high stability during hardening, high compressive strength, and good resistance to tempering back. This alloy's special qualities have made it valuable across various sectors. Die, trimming, coining, punching, shear blades, fuller, Phillips head forming dies, thermosetting resin forming dies, cold forming dies, fine blanking, stripper plates, brick molds, chisels, pneumatic tools, deep drawing and forming dies, cold drawing punches, hobbing, blanking, lamination and stamping dies, shear blades, burnishing rolls, master tools and gauges, slitting cutters, thread rolling & wire is just a few of the many uses for AISI D2^2–4^. It was observed that the dielectric oil waste generated at the end of the EDM operation was very toxic and could not be reused, making the EDM process unsustainable. The use of vegetable-based dielectrics offers significant advantages in terms of sustainability, as they are environmentally and operator-friendly and provide green and sustainable alternatives to hydrocarbon and water-based dielectric fluids^5,6^. Researchers have been experimenting with alternative dielectrics to improve process performance, reduce environmental impact, minimize fire hazards and increase operator safety. Canola, mineral oil, Sunflower, Olive oil, Soybean oil, Cotton seed oil, Grape seed oil, Rice bran oil, Neem oil, Peanuts oil, Palm oil and Waste Vegetable oil were used as dielectrics and compared to conventional dielectrics in terms of tool wear and material removal rates^6–16^. It was pointed out from the experimental results that vegetable and bio-oil-based dielectrics have great potential to replace traditional dielectrics, thus providing a more sustainable manufacturing process in the future. Tapas^17^ checked the Feasibility of jatropha oil as a dielectric fluid. Dissolved gas analysis proved that transesterified Jatropha oil provides sustainable and biodegradable dielectrics for EDM. In order to improve the sustainability of the EDM process, Jatropha crucas oil is used in the present work. Its seed contains nearly 30–40% oil by weight and has excellent thermal, mechanical and chemical properties. Jatropha crucas oil is non-edible; it does not affect the food chain due to its highly toxic tokialbumin cousin.

In powder-mixed EDM (PMEDM), the powder is mixed with dielectric to enhance the EDM surface. Gurpreet^18^ studied the effect of nano nano-size TiO_2_ powder mixed EDM on stainless steel 316 L; from the results, the addition of TiO_2_ powder contributed towards the superior surface finish (i.e., 0.266 µm). Houriyeh Marashi^19^ added Ti nanopowder to study the effects on the AISI D2 steel surface. It was reported that surface roughness considerably improved for all the machining conditions. Further, it also enhanced the morphology of the D2 steel surface as a result of a shallower crater and less formation of low-height ridges. Furthermore, surface micro-defects and cracks diminished. TiO_2_ powder helps with surface modification^20^. In the present study, TiO_2_ powder is mixed with the dielectric to reduce the surface's roughness, thus enhancing the surface's finish.

Table 1 shows the Use of ML algorithms in EDM. It was observed that S.R. can be predicted using an ML algorithm, so the optimum level of process parameters can be determined in advance, further reducing cost and time and also loss of labor^23–30^. However, more articles focus on implementing ML algorithms to predict the S.R. of EDM processes. Machine part quality is determined and evaluated by the feature of S.R. According to Wit Grzesik^31^, the basic characteristics of surface integrity are surface roughness/surface topography, certain metallurgical and microstructural changes, and process-induced residual stresses. According to Viktor P. Astakhov^32^, the surface integrity of a surface can be defined as a set of different characteristics (both the surface and the depth of the engineering surface, which influence the performance of this surface in use). These characteristics primarily include surface finish, texture, profile, fatigue corrosion and wear resistance, adhesion and diffusion properties. Surface integrity and surface texture are related to S.R., which defines the geometry of the workpiece surface^33^. Determination of S.R. by an analytic equation is quite difficult because of the complexity of surface roughness formation.

**Table 1:** Use of machine learning (ML) in the EDM process.

References	Year	Machined materials	Input parameters	Response parameters	Prediction method
^23^	2022	NiTi alloys, NiCu alloys and BCu alloys	Ton, Toff, Gap current and gap voltage	MRR	RF, D.T., Gradient Boosting ANN
^24^	2020	Aluminum	Voltage, Ton, Wire feed, dielectric pressure	S.R	Support vector method (SVM), Extreme learning machine, Weighted Extreme learning machine
^25^	2022	Shape memory alloys (SMA) Nitinol rods	Ton, Toff and current	S.R	AlexNet, KNN, MNB and DenseNet
^26^	2022	Memory alloy of Cu-based shape	Peak current (Ip), Ton, gap voltage and Toff	D.D. and TWR	GA and TLBO techniques
^27^	2021	Inconel 718	wire feed rate, Ip, Ton, Toff and servo voltage	SR	ANN, SVM
^28^	2021	EN31 tool steel	Ton, Toff, Ip, Vg, flushing pressure (P)	Tool shape prediction and S	.R DT, R.F., linear model and ANN
^29^	2020	Hastelloy C-276	Wire tension, flushing pressure, Ton, Toff, servo voltage and wire feed rate	Kerf width and S.R	Gradient descent method
^30^	2022	EN31 steel	Discharge current (Ip), gap voltage, Ton, Toff and flushing pressure (P)	S.R. and prediction of tool shape	Linear regression

The present study is carried out to verify the operational and technical feasibility of Jatropha oil as a dielectric in the EDM process. Surface roughness values are measured during the machining of AISI D2 steel using commercially available EDM oil and Jatropha oil as a dielectric. To enhance the S.R. of the EDMed surface, titanium dioxide powder is mixed with the dielectrics. S.R. is predicted using different machine learning methods like Linear Regression, Decision Tree and Random forest. Further evaluation metrics were used to analyze the performance of each model.

## Materials and methods

Electronica makes Die sink type EDM machine used to perform this experiment, model C400*250, having negative polarity to the electrode. The workpiece material used in the study is AISI D2 steel of size 10 × 10 × 10 mm. The chemical composition of AISI D2 tool steel is (1.55% C, 0.3%Si, 0.4%Mn, 11.8% Cr, 0.8% Mo, and 0.8% V)^19^. Due to its high electrical conductivity, an electrode of copper material is selected with a radius of 12.5 mm. The EDM is carried out on the workpiece using two variants of dielectric fluid, i.e., Jatropha oil and commercially available EDM oil. The titanium dioxide (TiO_2_) nanoparticles are suspended in the dielectric fluid. The machining tank consists of a small acrylic tank that adds titanium oxide to the dielectric fluid. The powder is constantly stirred with a Kenwood (250 W) stirrer so that the powder gets uniformly disturbed and does not accumulate at the bottom of the tank. Mitutoyo surftest (SJ 201) measures S.R. using different process parameters like current, gap voltage, pulse on time and pulse off time (4 levels of each) as depicted in Table 2. Three readings are taken for S.R. measurement at different points than the average value is selected. Experiments have been performed using the OVAT approach. A total of 20 sets of experimental trials have been performed for each dielectric fluid, having 10 min of machining time for each set. Table 3 shows the properties of EDM and Jatropha oil. Table 4 shows S.R.'s Experimental layout and observed values using commercially available EDM oil and jatropha oil.

**Table 2:** Experimental process parameters and their levels.

Parameters	Levels	Machine setting available	Constant
Pulse current (A)	3, 6, 9, 12, 15	0–40 Amperes	9
Pulse ON time (μs)	30, 50, 100, 200, 400	10 steps	100
Pulse OFF time (μs)	4, 6, 8, 10, 12	10 steps	12
Gap voltage (V)	50, 55, 60, 65, 70	0–200 V	50

**Table 3:** Properties of EDM oil and Jatropha oil^12,13^.

Sr. No.	Properties	EDM Oil	Jatropha oil
1	Density (gm/ml)	0.775	0.870
2	Viscosity at (27 °C) (cSt)	2.33	6.5836
3	Thermal conductivity (W/m K)	0.139	0.147
4	Specific heat (kJ/kgK)	1.95	1.90
5	Breakdown voltage (kV)	56	26
6	Dielectric constant at (27 °C)	2.02	3.238
7	Flash point ( °C)	108	170
8	Oxygen content (wt.%)	0.070	1.11
9	Carbon content (wt.%)	94.9	85.32

**Table 4:** Experimental layout and observed values of S.R.

Sr. No. of sample	Current (A)	Pulse on time (μs)	Pulse off time (μs)	Gap voltage (V)	EDM oil	Jatropha oil
					SR (μm)	SR (μm)
1	9	30	12	50	4.58	4.5
2	9	50	12	50	5.55	6.61
3	9	100	12	50	6.36	8.82
4	9	200	12	50	7.5	11.13
5	9	400	12	50	8.23	12.24
6	3	100	12	50	5.15	4.627
7	6	100	12	50	6.88	6.66
8	9	100	12	50	8.25	8.46
9	12	100	12	50	10.28	9.22
10	15	100	12	50	11.24	9.73
11	9	100	12	50	8.64	7.77
12	9	100	12	55	8.07	8.44
13	9	100	12	60	7.38	8.26
14	9	100	12	65	7.74	7.94
15	9	100	12	70	7.72	7.64
16	9	100	4	50	6.39	7.12
17	9	100	6	50	8.38	7.12
18	9	100	8	50	8.45	7.65
19	9	100	10	50	8.83	8.77
20	9	100	12	50	8.5	9.17

### Machine learning (ML) techniques

**Figure 1:**
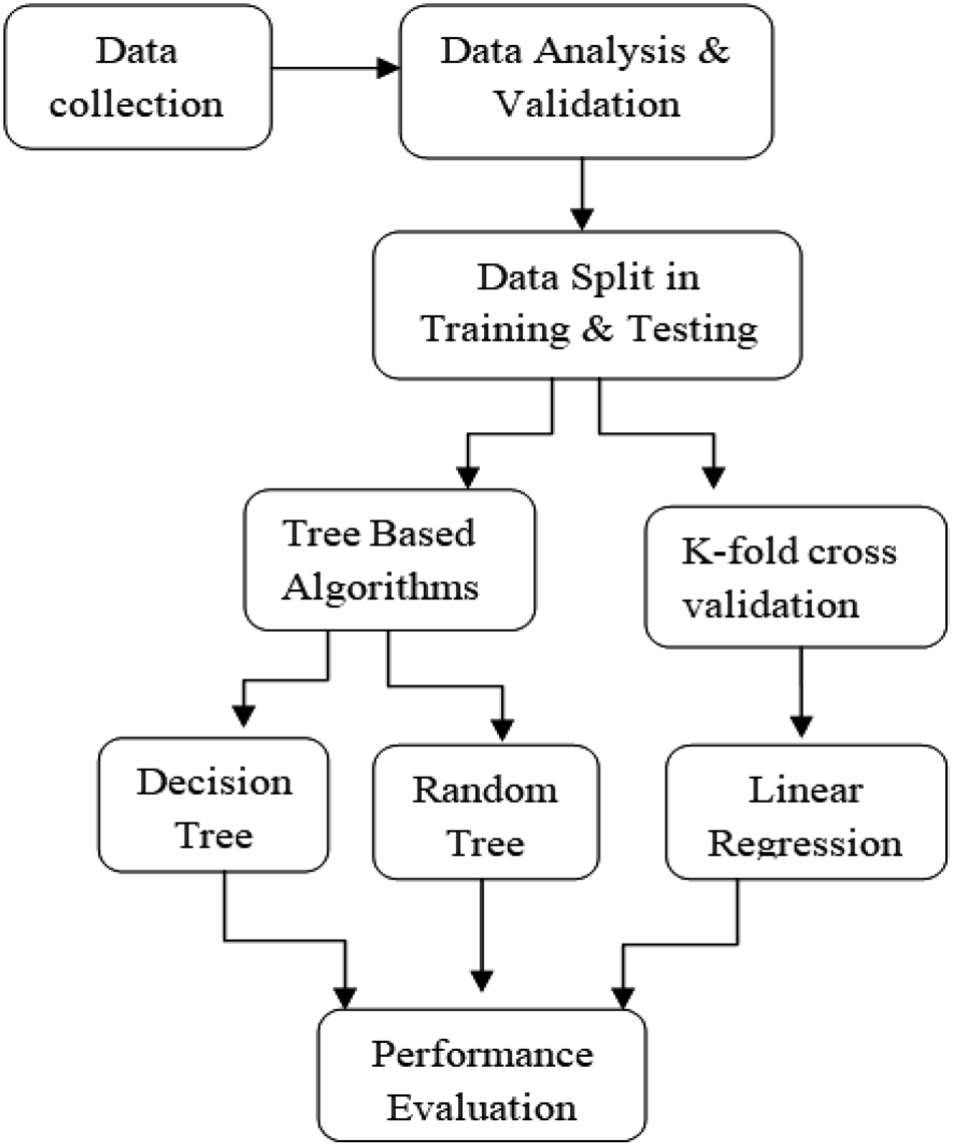
Flowchart for implementation of ML algorithms.

In the present work, three ML algorithms, i.e., Linear regression (L.R.), Decision Tree (D.T.) and Random forest (R.F.), were used to predict S.R. Figure 1 shows a flowchart for the Implementation of ML algorithms on the experimental datasets. Linear Regression (L.R.) establishes a linear relation between dependent and independent variables. L.R. provides better solutions to each problem, making its interpretation and implementation easy. It is only applicable for linear solutions, so that provides a limit to it. If you have many features in a small dataset with less distortion, L.R. may outperform D.T. and R.F. algorithms^33–38^. Random Forest Regression (R.F.) is a supervised ML model built using multiple decision trees at random, where the number of votes of the forest is selected as the prediction output. R.F. models provide more accurate, robust, and reliable predictions than a single decision tree. In addition, random forest models are less prone to overfitting than decision trees^33–38^.

Decision tree Regression (D.T.) is a supervised ML predictive model in which information is split continuously based on specific parameters. In the Decision tree regression model, its branches denote the decision rules; internal nodes denote features of input data provided, and leaf nodes show the outcome. It is the same as a tree structure^23^. D.T. models do not require preprocessing of data and can efficiently handle collinearity. In general, decision tree models have better average accuracy. D.T. models are prone to outliers and may lose valuable information during handling continuous variables^33–38^.

### Performance evaluation metrics (PEM)

It is used to analyze regression-based ML models' performance^24^. Table 5 defines the equations and significance of each Performance Evaluation Metrics. Each of these metrics provides information from a different perspective about the performance of the ML model.

**Table 5:** Equations and significance of performance evaluation metrics.

Performance metrics	Equations	Significance
Absolute mean error (AME)	$\sum_{{\text{i}}=1}^{{\text{n}}}\frac{y{\text{i}}-\stackrel{`}{y}{\text{i}}}{{\text{n}}}$	It measures the prediction error and does not show the direction of the error, i.e., whether it is over or under-predicting the data
Squared mean error (SME)	$\sum_{{\text{i}}=1}^{{\text{n}}}\frac{(y{\text{i}}-\stackrel{`}{y}{\text{i}}{)}^{2}}{{\text{n}}}$	Measures fitness of the model. It penalizes even small errors by squaring them, leading to overestimating errors
Root mean squared error (RMSE)	$\sqrt{\sum_{{\text{i}}=1}^{{\text{n}}}\frac{(y{\text{i}}-\stackrel{`}{y}{\text{i}}{)}^{2}}{{\text{n}}}}$	Measures the average error performed by the model. It handles the penalization of smaller errors done by MSE by square rooting, hence prone to outliers
Regression (R-square coefficient)	$\frac{1-\sum_{{\text{i}}=1}^{{\text{n}}}(y{\text{i}}-\stackrel{`}{y}{\text{i}}{)}^{2}}{\sum_{{\text{i}}=1}^{{\text{n}}}(y{\text{i}}-\overline{y }{)}^{2}}$	Measures the performance of your model. It shows how well the model fits the dependent variables

## Results and discussion

### Effects of various process parameters on S.R.

**Figure 2:**
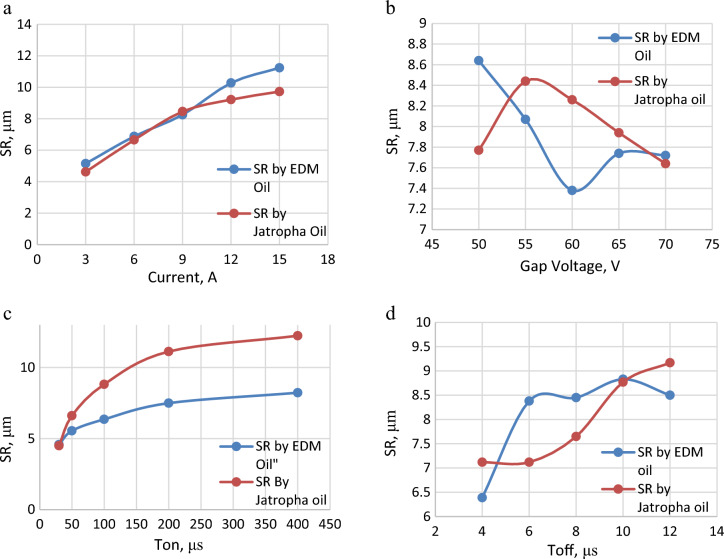
(**a**) Effect of current on SR. (**b**) Effect of gap voltage on SR. (**c**) Effect of ton on SR. (**d**) Effect of Toff on SR.

**Figure 3:**
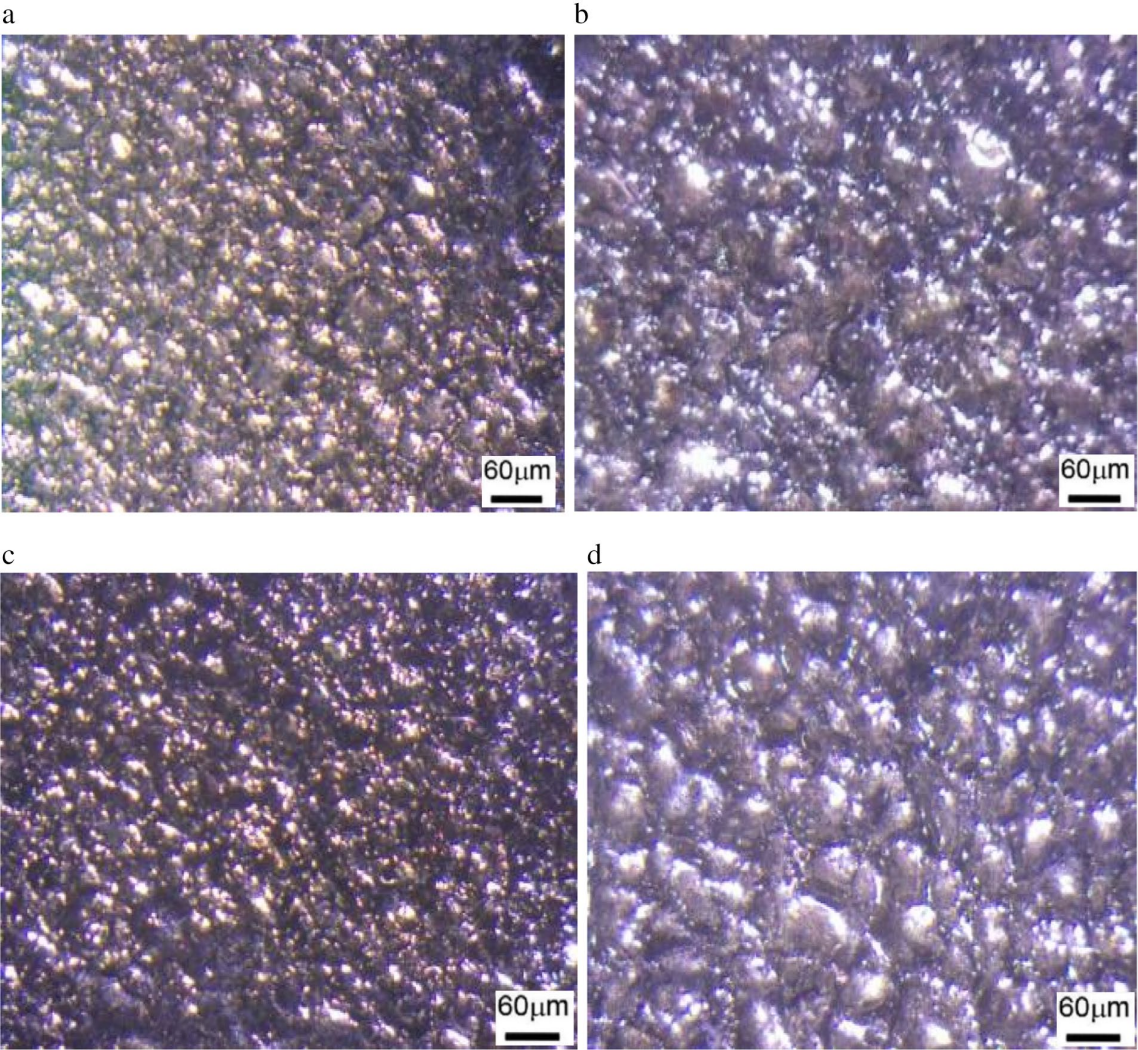
(**a**) Surface morphology under (current = 9A, Ton = 30 μs, Toff = 12 μs and Gap voltage = 50 V) using EDM oil as a dielectric. (**b**) Surface morphology under (current = 15A, Ton = 100 μs, Toff = 12 μs and Gap voltage = 50 V) using EDM oil as a dielectric. (**c**) Surface morphology under (current = 9A, Ton = 30 μs, Toff = 12 μs and Gap voltage = 50 V) using Jatropha oil as a dielectric. (**d**) Surface morphology under (current = 9A, Ton = 400 μs, Toff = 12 μs and Gap voltage = 50 V) using Jatropha oil as a dielectric.

The influence of various process parameters like Gap current, Gap voltage, Pulse ON time (Ton) and Pulse OFF time (Toff) on S.R. have been investigated and discussed below. Table 4 shows Experimental Data. Surface Roughness is associated with an average roughness of the surfaces produced. The lower value of S.R. is desirable as it is directly associated with the part quality. Figure 2a–d shows the effect of various process parameters on the S.R. Figure 2a shows the influence of current on S.R. It was observed that as current increases, the surface roughness value also increases for both dielectric fluids. Figure 3b depicts surface morphology at machining conditions (current = 15 A, Ton = 100 μs, Toff = 12 μs and Gap voltage = 50 V), yielding S.R. of 11.54 microns using EDM oil as a dielectric fluid. The high value of S.R. is observed at the maximum value of Current. The spark discharge expands, as the pulse current to the work material, and the collision force generated in the crater increases the strength at high Current, affecting more work materials. Furthermore, the increased energy from the increased current penetrates the material surface, eventually forming deeper and wider craters and resulting in a rough surface^11–14^. It was also observed that using jatropha oil as a dielectric yields similar results to EDM oil. Figure 2b shows the influence of gap voltage on S.R. It was observed that gap voltage had a limited influence on the S.R.

**Figure 4:**
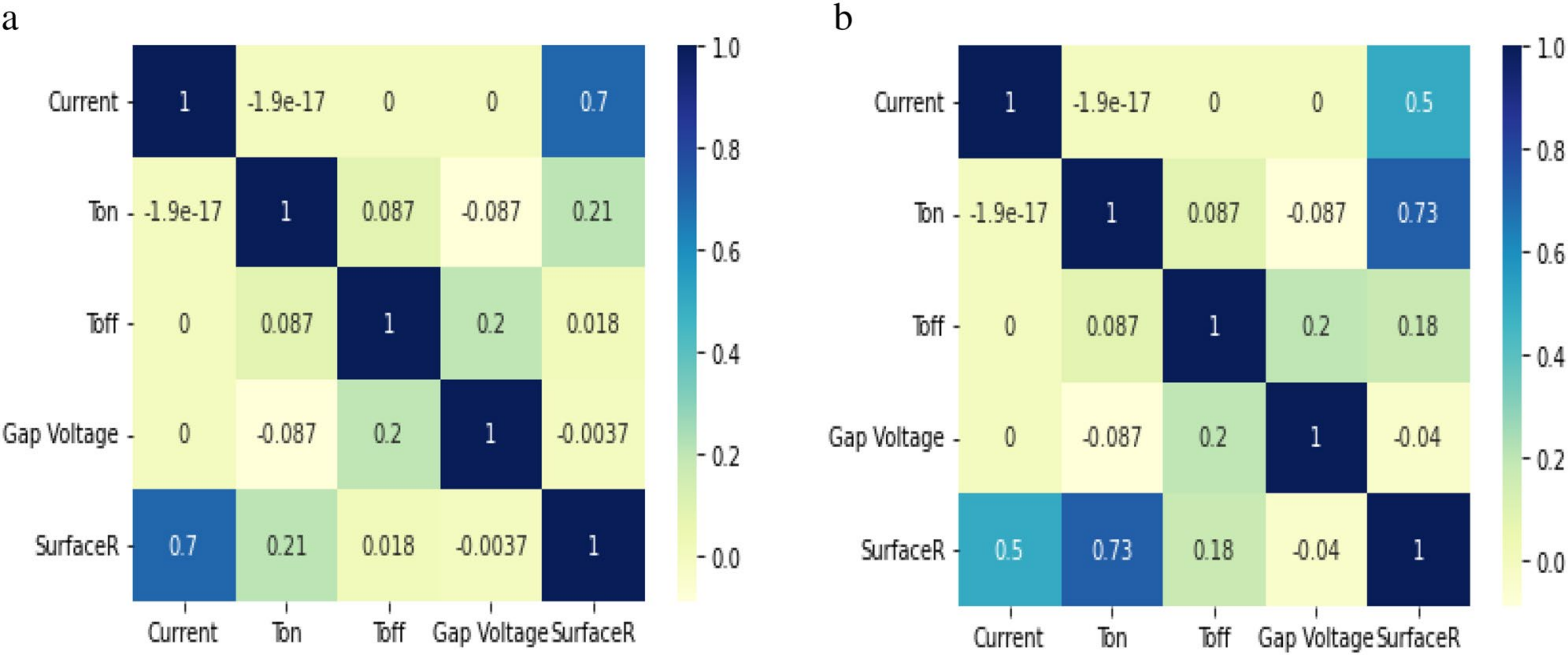
(**a**) Correlation heat map into responses and process parameters for EDM oil. (**b**) Correlation heat map into responses and process parameters for Jatropha Oil.

Figure 2c) shows the influence of pulse on time (Ton) on S.R. S.R. increases as Ton increases for both the dielectric fluid. Optical micrographs are taken at 200 X magnificent. Figure 3a) depicts surface morphology at machining condition (current = 9A, Ton = 30 μs, Toff = 12 μs and Gap voltage = 50 V) using EDM oil as a dielectric which yield S.R. of 4.508 microns, which is the lowest value of S.R. measured and Fig. 3c) depicts surface morphology at machining condition (current = 9A, Ton = 30 μs, Toff = 12 μs and Gap voltage = 50 V) using Jatropha oil as a dielectric which yield S.R. of 4.5 microns. It was observed that both dielectric at the lowest levels of Ton (i.e., 30) minimum value of S.R. (i.e., 4.5 microns) is achieved. Further, Fig. 3d) depicts surface morphology at machining conditions (current = 9A, Ton = 400 μs, Toff = 12 μs and Gap voltage = 50 V) using Jatropha oil as a dielectric, which yields S.R. of 12.24 microns, which shows that the value of S.R. goes on increasing as levels Ton increases. At the same time, other process parameters are kept constant. This is attributed to more prolonged sparking time causing more prolonged melting and vaporization, which causes deeper and wider crater formation resulting in higher value of S.R., also can be seen from the surface morphology of the specimen^11–14^. Figure 2d) shows the influence of Toff on S.R. It was observed that as the interval of Toff increases, the value of S.R. also increases. Also, from Fig. 4a,b Correlation heat map between the Toff and S.R., it can be seen that Toff has less significance on S.R. for both dielectric fluids. Figure 4 shows a correlation heat map for the process parameters with S.R. obtained using EDM oil and jatropha oil. Using EDM oil as dielectric, S.R. obtained was noticed to have a moderately strong positive correlation with gap current, Ton, mildly related Toff and be negatively correlated with gap voltage. Using jatropha oil as dielectric, S.R. obtained was noticed to have a somewhat positive correlation with Ton, gap current, mildly related Toff and be negatively correlated with gap voltage. It is observed that Gap current and Ton significantly influence the S.R. in the case of both dielectric fluids.

### Prediction of S.R. using linear regression (L.R.)

During Linear Regression the dataset was normalized further; it was split into a set of training and testing in which a dataset of training was used to construct a model and for evaluation testing dataset was utilized. The regression model for S.R. prediction using oil of EDM and Jatropha is shown in Eqs. (1) and (2), respectively.

1 $$\begin{gathered} {\text{S}}.{\text{R}} = {1}.{3433} + 0.{\text{4683 x}}_{{1}} + 0.00{\text{45x}}_{{2}} + 0.0{\text{142 x}}_{{3}} + 0.0{\text{233x}}_{{4}} \hfill \\ {\text{R}}^{{2}} = 0.{611} {\text{Adj R}}^{{2}} = 0.{438} \hfill \\ \end{gathered}$$

2 $$\begin{gathered} {\text{S}}.{\text{R}} = 0.{5}0{62} + .0.{\text{4186 x}}_{{1}} + 0.0{\text{182 x}}_{{2}} + 0.0{\text{891 x}}_{{3}} + 0.0{\text{119 x}}_{{4}} \hfill \\ {\text{R}}^{{2}} = 0.{825} {\text{Adj R}}^{{2}} = 0.{748} \hfill \\ \end{gathered}$$

where, x_1_ is current, x_2_ is Ton, x_3_ is Toff and x_4_ is Gap voltage.

The R^2^ values showed that the linear regression model using Jatropha oil captured 82% of the variance in the target variable, while for EDM oil, only 61%.

### Prediction of S.R. using decision tree (D.T.)

**Figure 5:**
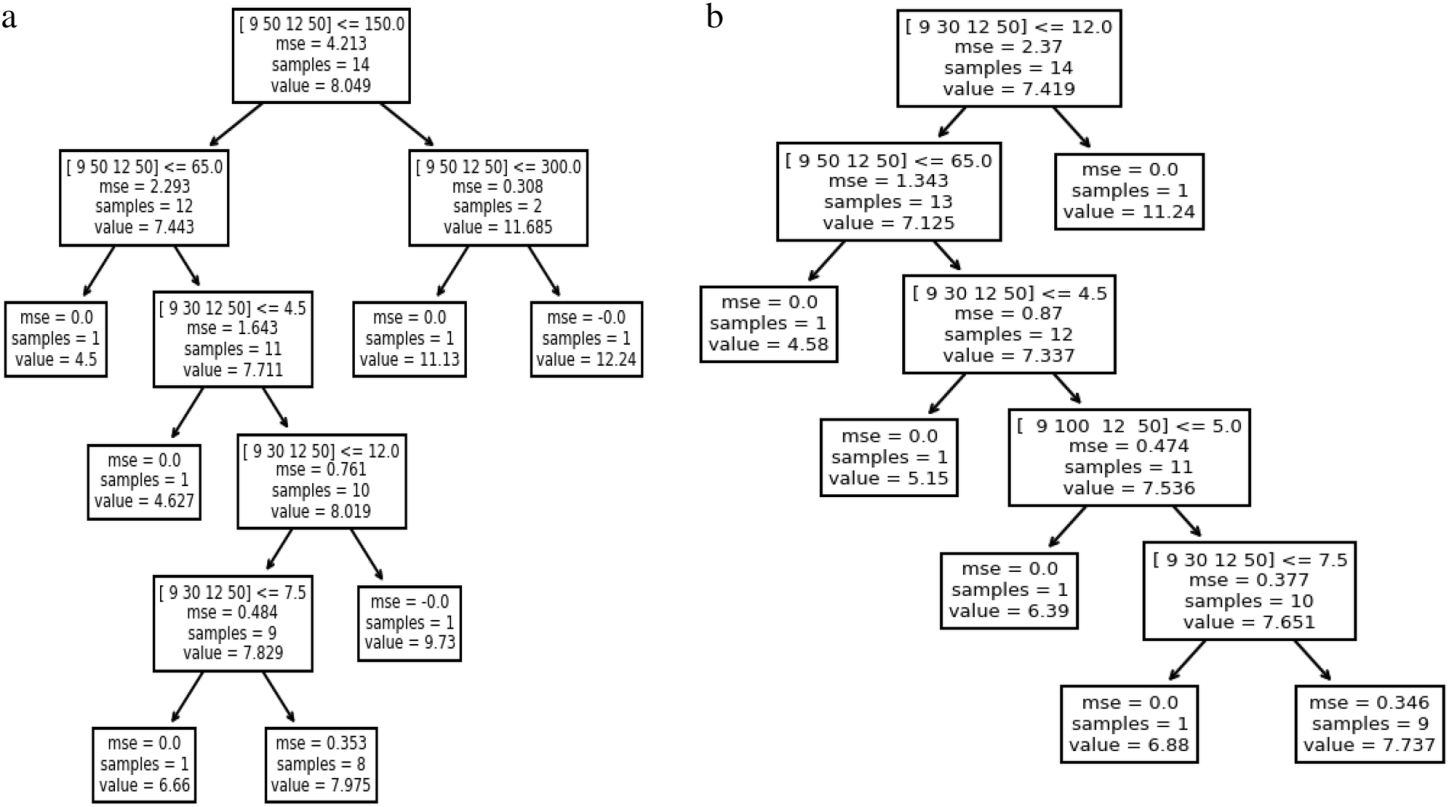
(**a**) Decision Tree Regression plot using EDM oil as a dielectric. (**b**) Decision Tree Regression plot using Jatropha oil as a dielectric.

The Decision Tree algorithm dataset of 20 samples was randomly split into training sets where 7 and 14 samples were selected for testing. Figure 5a,b show the obtained Decision Tree plot using EDM and Jatropha, respectively.

From the R^2^ values, it was observed that the regression model using Jatropha oil captured 82% of the variance in the target variable, while EDM oil captured only 72%. It was also noticed that decision tree algorithms give a lower value of Mean Squared Error and Mean Absolute Error value using Jatropha oil than EDM oil, as seen in Table 6.

**Table 6:** PEM using decision tree.

Sr. No.	Evaluation metrics	Values with EDM oil metrics	Values with Jatropha oil
1	Mean absolute error	1.16	0.97
2	Mean squared error	1.75	1.36
3	Root mean squared error	1.3	1.16
4	Regression (R^2^) coefficient	0.72	0.82

### Prediction of S.R. using Random Forest (R.F.)

Table 7 shows Performance Evaluation Metrics using the Random Forest regression model for EDM and Jatropha oil. From the R^2^ values, it was observed that the regression model using Jatropha oil captured 89% of the variance in the target variable, while for EDM oil, only 55%. It was also noticed that the random forest algorithm gives a lower value of Mean Squared Error and Mean Absolute Error value using Jatropha oil than EDM oil.

**Table 7:** Performance evaluation metrics using Random Forest.

Sr. No.	Evaluation metrics	Value with EDM oil	Value with Jatropha oil
1	Mean absolute error	1.39	0.61
2	Mean squared error	2.70	0.44
3	Root mean squared error	1.64	0.66
4	Regression (R^2^) coefficient	0.55	0.89

Shanmugasundar^36^ carried out a comparative analysis of Linear, Random Forest, and AdaBoost Regressions where regression models were developed for MRR, EWR and S.R. For S.R., the value of R^2^ by L.R. model predicted was only 56.8% and for R.F. regression model 86.5%. Jatti^23^ predicted the value of MRR using four supervised machine learning regression models: Random Forest, Decision Tree, Gradient Boosting and Artificial Neural Network. For MRR, the value of R^2^ by the D.T. model predicted was only 0.814, and the R.F. regression model was 0.856. Based on the comprehensive evaluation, it is observed that although L.R. is simple and quick, it needs to be more adequate to explain all the variances in the dataset and can accurately map the complex relationship between the process parameters and responses. R.F. regression was found to be suitable for predictive modeling.

Table 8 shows the predicted R^2^ for each ML method using commercially available EDM oil and Jatropha oil. R.F. algorithm provides better results than D.T. and L.R. using jatropha as a dielectric media. From the R^2^ values, it was observed that regression models using Jatropha oil were able to capture more variance in the target variable when compared with EDM oil. R.F. model provides S.R. with a R^2^ value of 0.89 and gives excellent performance. This study shows that machine learning methods can forecast the S.R. of AISI D2 tool steel with EDM.

**Table 8:** Performance evaluation metrics of regression-based models.

Sr. No.	Model	R^2^ value with EDM oil	R^2^ value with Jatropha oil
1	LR (Linear Regression)	0.61	0.82
2	DT (Decision Tree)	0.72	0.82
3	RF (Random Forest)	0.55	0.89

### Analysis of variance

This section explains the analysis of variance for the surface roughness considering EDM oil and Jatropha oil separately. Table 9 depicts the ANOVA for SR-Jatropha oil. Considering level of significance (α) as 0.05, any value less than 0.05 is considered as most significant parameter. Table 9 showed that gap current and pule on time are most significant parameter that affects the surface roughness.

**Table 9:** Analysis of variance for SR-Jatropha oil, using adjusted SS for tests.

Source	DF	Seq SS	Adj SS	Adj MS	F	P
Ig	4	18.3060	18.8600	4.7150	13.17	0.030
Ton	4	41.2406	40.4275	10.1069	28.24	0.010
Toff	4	2.9333	3.3995	0.8499	2.37	0.252
Vg	4	0.8452	0.8452	0.2113	0.59	0.695
Error	3	1.0737	1.0737	0.3579		
Total	19	64.3989				
S = 0.598247			R-Sq = 98.33%		R-Sq (adj) = 89.44%	

Table 10 depicts the ANOVA for SR-EDM oil. Considering level of significance (α) as 0.1, any value less than 0.1 is considered as most significant parameter. Table 10 showed that gap current is the most significant parameter that affects the surface roughness.

**Table 10:** Analysis of variance for SR-EDM oil, using adjusted SS for tests.

Source	DF	Seq SS	Adj SS	Adj MS	F	P
Ig	4	26.794	24.877	6.219	5.49	0.097
Ton	4	14.690	12.913	3.228	2.85	0.208
Toff	4	3.714	3.639	0.910	0.80	0.596
Vg	4	0.326	0.326	0.082	0.07	0.986
Error	3	3.396	3.396	1.132		
Total	19	48.921				
S = 1.06397			R-Sq = 93.06%		R-Sq (adj) = 86.03%	

**Figure 6:**
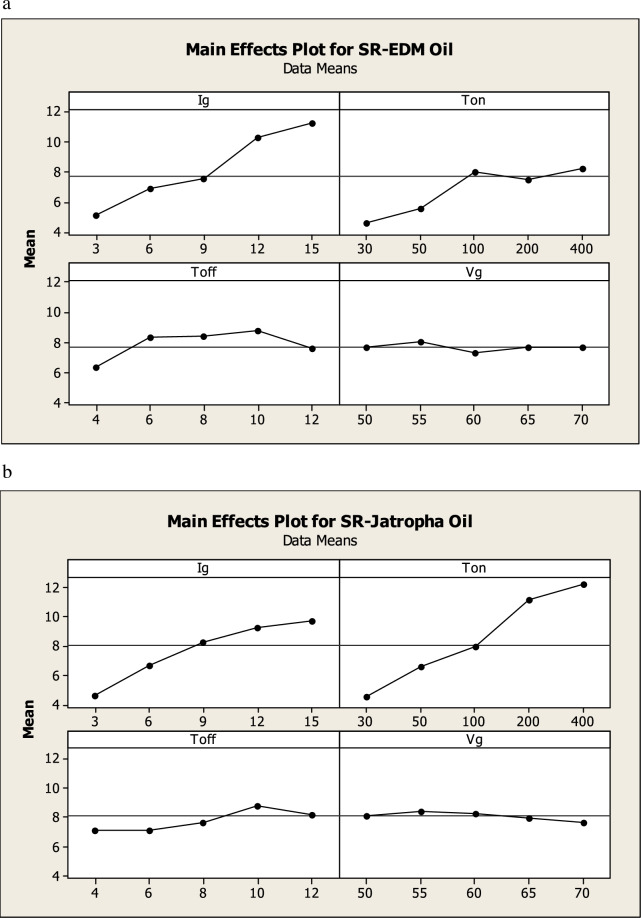
(**a**) Effect of EDM input process parameters on SR using EDM oil. (**b**) Effect of EDM input process parameters on SR using Jatropha oil.

Figure 6a,b shows the trend line for SR with respect to EDM process parameters. Figure 6a shows that gap current is most significant parameter followed by pulse on time, pulse off time and gap voltage. Figure 6b depicts that gap current and pulse on time are the most significant parameter.

## Conclusion

The present study depicts the utilization of Jatropha oil as dielectric to technically and operationally feasible aspects of the EDM process. Results obtained using Jatropha oil were almost in line with those obtained using commercially available EDM oil.

- Response trends of S.R. using Jatropha oil under the influence of process parameters are similar to those of EDM oil, which proves Jatropha oil is a technically and operationally feasible dielectric.
- For EDM and Jatropha oil, the lowest value of S.R. (i.e., 4.5 microns) was obtained at current = 9, Ton = 30, Toff = 12, and gap voltage = 50. It was found that the S.R. increases along with the current and Ton values. Current and Ton was observed to be the most important factor influencing S.R.
- For predicting S.R., supervised ML algorithms based on regression models were implemented. Random forest modeling is highly accurate, with an R^2^ value of 0.89 and an MSE of 1.36% among all methods. Subsequently, linear regression and decision tree models also show good accuracy, with an R^2^ value of 0.82.
- Random forest models have better predictive capabilities and may be one of the best options for modeling complex EDM processes.
- ML algorithms can analyze and model EDM processes, potentially replacing time-consuming and costly experiments.

Future scope will be to investigate how jatropha oil as a dielectric can improve the sustainability of the EDM process. Also, more ML modeling and simulation research is needed to analyze material removal rate, crater depth, surface crack density, and residual stress.

## Data Availability

The data that supports the findings of this study are available within the article.
